# Bilateral Choroidal Detachment Induced by Unilateral Application of a Fixed Combination of Topical Timolol Maleate and Brinzolamide

**Published:** 2016

**Authors:** Oya DONMEZ, Hilal KILINC, Zeynep OZBEK, Ali Osman SAATCI

**Affiliations:** 1 Specialist, Department of Ophthalmology, TC Hitit University, Izmir, Turkey; 2 Resident, Department of Ophthalmology, Dokuz Eylul University, Izmir, Turkey; 3 Professor, Department of Ophthalmology, Dokuz Eylul University, Izmir, Turkey

**Keywords:** Choroidal Detachment, Brinzolamide, Timolol Maleate, Phacoemulsification, Acetazolamide, Visante

## Abstract

We describe a 66-year-old man who developed bilateral choroidal detachment that was induced by unilateral topical administration of a fixed combination of 1% brinzolamide and 0.5% timolol maleate the day after an uneventful phacoemulsification surgery and intraocular lens implantation involving his right eye. We believe that the reaction was an idiosyncratic reaction, most likely against brinzolamide. The condition improved rapidly after the cessation of the fixed combination of brinzolamide and timolol maleate and treatment with 1% topical prednisolone acetate every hour and 1% cyclopentolate twice a day bilaterally. Although there are several similar cases involving choroidal detachment after oral acetazolamide and topical dorzolamide treatment mentioned in the literature, the present case is the first case report involving bilateral choroidal detachment after topical treatment with brinzolamide.

## INTRODUCTION

 Choroidal detachment (involving ciliochoroidal detachment, uveal effusion, or ciliochoroidal effusion) is characterized by the accumulation of abnormal fluid in the suprachoroidal space (1-2). There are several case reports describing ciliochoroidal effusion (occasionally with acute angle-closure glaucoma) following treatment with oral acetazolamide or topical dorzolamide (3-8). However, this is the first case report on bilateral choroidal detachment after topical treatment with brinzolamide, a carbonic anhydrase inhibitor derived from a novel class of heterocyclic sulfonamides with high affinity and inhibitory activity against human carbonic anhydrase II (9)

## CASE REPORT

 A 66-year-old man was referred to us for the treatment of bilateral choroidal detachment following an uneventful unilateral phacoemulsification surgery and intraocular lens (IOL) implantation in his right eye. His right eye was treated with a fixed combination of topical 0.5% timolol maleate and 1% brinzolamide twice a day and 1% prednisolone acetate six times a day, beginning the morning after the surgery. He started to experience visual deterioration first in the right eye than in the left eye later on the same day. 

**Figure 1:**
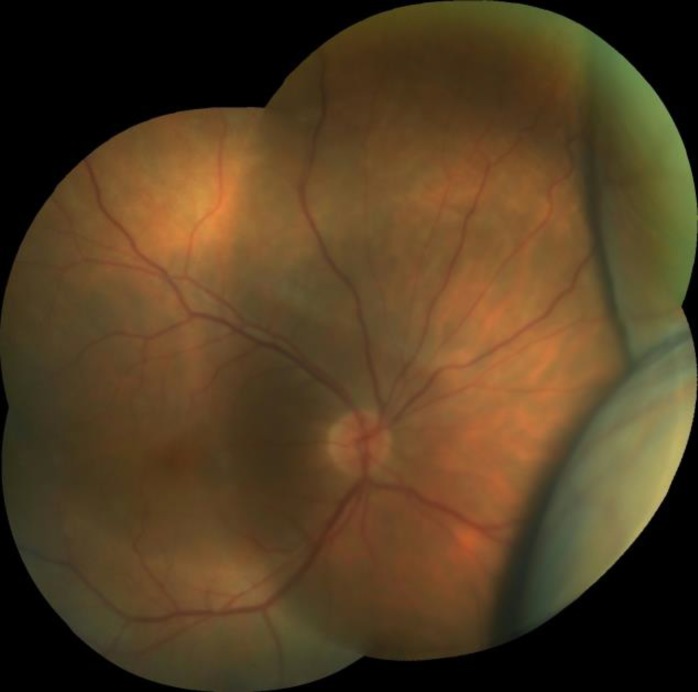
Color photograph of the right eye, obtained four days after the unilateral phacoemulsification surgery and IOL implantation, showing extensive choroidal detachment.

**Figure 2:**
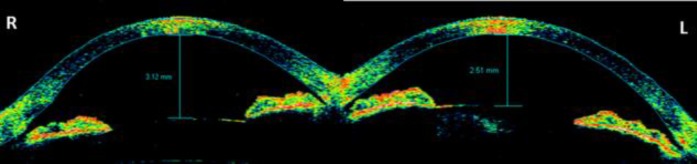
Anterior segment optical coherence tomography image showing the anterior chamber depth in the right and left eyes at initial presentation.

An examination on the fourth day after the surgery showed that the patient’s visual acuity was 4/10 in in the right eye (-1.0 x 90) and 7/10 in the left eye (+0.75). A slit-lamp examination revealed mild ciliary injection and a slightly shallow anterior chamber in both eyes. In the right eye, there were a moderate number of cells in the anterior chamber (grade 2+) and the IOL was well-centered in the bag, while the left eye had grade 2+ nuclear sclerosis. The intraocular pressure (assessed using an applanation tonometer) was 10 mmHg in the right eye and 20 mmHg in the left eye. A dilated fundus examination revealed bilateral 360° annular choroidal detachment, which was more pronounced in the right eye (i.e., the eye that was operated on) (Figure 1). No pertinent past medical history was presented relevant to the formation of the bilateral choroidal detachment. The choroidal detachment in the left eye was detected using a Goldmann three-mirror lens. Optical coherence tomography (OCT) images indicated that both maculae were normal. The axial lengths were 23.12 and 23.09 mm in the right and left eye, respectively, as assessed using an IOL Master 500 (software version 2.02, Carl Zeiss, Jena, Germany). The anterior chamber depth was 3.12 mm in the right eye and 2.51 mm in the left eye, based on anterior segment OCT (Visante OCT system, Model 1000, software version 3.0, Carl Zeiss AG) (Figure 2). 

We believe that the bilateral choroidal detachment was most likely an idiosyncratic reaction to the brinzolamide. The fixed combination of brinzolamide and timolol maleate was discontinued, and 1% prednisolone acetate was administered bilaterally every hour, while 1% cyclopentolate was administered bilaterally twice a day. 

**Figure 3:**
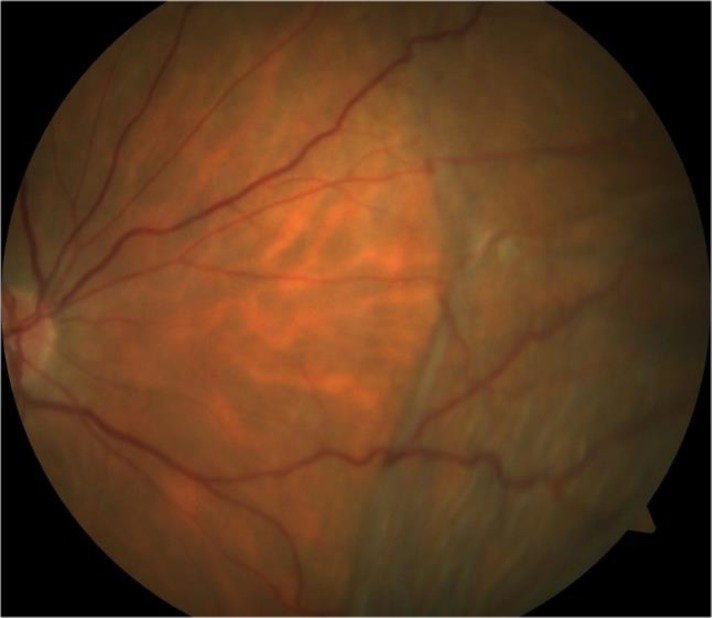
Color photograph of the right eye, obtained 3 days after the discontinuation of the fixed combination of 0.5% timolol maleate and 1% dorzolamide, showing marked resolution of the original choroidal detachment.

**Figure 4:**
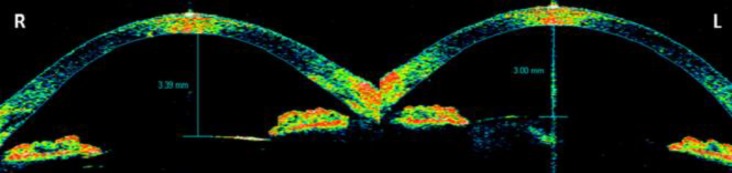
Anterior segment optical coherence tomography images, 3 days after the discontinuation of the fixed combination of 0.5% timolol maleate and 1% dorzolamide, showing increased anterior chamber depth in the right and left eyes.

Three days after treatment with fixed combination of brinzolamide and timolol maleate was discontinued, the patient’s visual acuity was 20/25 in the right eye and 20/20 in the left eye. The intraocular pressure was 16 mmHg in the right eye and 17 mmHg in the left eye. A fundus examination revealed that the choroidal detachment had markedly improved in the right eye (Figure 3) and almost totally cleared in the left eye. The anterior chamber depth had increased to 3.39 mm in the right eye and 3.00 mm in the left eye (as assessed using the Visante OCT system) (Figure 4).

## DISCUSSION

 It is known that oral acetazolamide and topical dorzolamide can cause ciliary body swelling, choroidal effusion, and forward displacement of the iris–lens diaphragm, most likely due to idiosyncratic reactions. Krieg and Schipper (10) suggested that the sulfonamide group of acetazolamide and dorzolamide stimulates the synthesis of prostaglandin E2, which in turn causes vasodilatation and increased vascular permeability in the anterior uvea. 

Several case reports have indicated that there is a causal relationship between oral acetazolamide and choroidal detachment. Mancino et al. (4) reported on a 76-year-old man who underwent cataract extraction and IOL implantation and developed bilateral angle-closure glaucoma with extensive choroidal detachment following the intake of two 250-mg doses of oral acetazolamide (the first was taken immediately after the surgery and the second was taken the next day). Interestingly, the same surgery was uneventfully performed on the patient’s other eye 7 years earlier. Cessation of acetazolamide and treatment with high-dose intravenous steroid therapy resulted in rapid clinical improvement. Malagola et al. (5) reported on a 71-year-old man who underwent routine unilateral cataract surgery and IOL implantation (the patient’s other eye was phakic). The patient took 250 mg oral acetazolamide 4 hours after the surgery and, 3 hours later, he experienced severe pain and bilateral visual deterioration. Both anterior chambers were shallow and the intraocular pressure was >50 mm Hg in both eyes. B-scan ultrasonography showed bilateral choroidal effusion. The patient discontinued the acetazolamide and, 3 days later, the intraocular pressure returned to normal and the myopic shift decreased. A month later, the patient underwent cataract surgery on the other eye without use of a sulfonamide medication, and no complications were noted. Parthasarathi et al. (5) reported on a 66-year-old man with chronic open-angle glaucoma who underwent unilateral cataract surgery with IOL implantation. He was treated with 250 mg oral acetazolamide immediately after the surgery and overnight. Three hours after the surgery, the patient developed bilateral angle-closure glaucoma and choroidal effusion. The condition resolved only after the acetazolamide was discontinued.

Like oral acetazolamide, topical dorzolamide may also induce choroidal detachment. Davani et al. (6) reported on a 70-year-old woman with ocular hypertension who had undergone bilateral extracapsular cataract extraction and posterior chamber IOL implantation 20 years previously. She was originally being treated with topical 0.05% timolol maleate but switched to a fixed combination of topical 0.5% timolol maleate and 1% dorzolamide. Five days after switching, she experienced bilateral visual deterioration and bilateral choroidal detachment. However, a further five days after cessation of the fixed combination of timolol maleate and dorzolamide, the choroidal detachment subsided in both eyes and treatment with 0.5% timolol maleate was re-initiated without any fundus changes. Goldberg et al. (7) reported a case of a 76-year-old patient with open-angle glaucoma and no history of previous ocular surgery who developed unilateral choroidal detachment a day after initiating treatment with 1% topical dorzolamide and 0.5% timolol maleate twice a day. Both drugs were discontinued and corticosteroid drops were administered, and the choroidal detachment resolved a week later. Doherty et al. (8) reported on a 75-year-old man who had undergone an uncomplicated phacoemulsification surgery with IOL implantation 7 years previously, and who had been using a fixed combination of latanoprost and timolol maleate for a long time for the treatment of chronic open-angle glaucoma. This regimen was supplemented with dorzolamide drops and, two days later, the patient’s visual acuity declined in both eyes. Fundoscopy revealed severe bilateral choroidal detachment without significant anterior chamber shallowing. The choroidal detachment resolved completely 2 weeks after the cessation of the topical dorzolamide.

Our case is the only reported case characterized by choroidal detachment and a slightly shallow anterior chamber in both eyes that is related to an idiosyncratic reaction to brinzolamide. Initiation and cessation of the drug showed a temporal relationship with the bilateral onset and resolution of the effusion, respectively. We recommend that clinicians consider topical brinzolamide (in addition to topical dorzolamide and oral acetazolamide) as a potential causative agent if choroidal detachment (particularly bilateral detachment) is detected after the administration of these drugs.
